# High Transmission All-Optical Combinational Logic Circuits Based on a Nanoring Multi-Structure at 1.31 µm

**DOI:** 10.3390/mi14101892

**Published:** 2023-09-30

**Authors:** Salma Ali Sadeq, Mohsen Hayati, Saba Khosravi

**Affiliations:** Electrical Engineering Department, Faculty of Engineering, Razi University, Tagh-E-Bostan, Kermanshah 6714414971, Iran; salmaali19917@gmail.com (S.A.S.); s.khosravi@razi.ac.ir (S.K.)

**Keywords:** IMI plasmonic waveguide, optical combinational logic gate, transmission spectrum, constructive and destructive interference

## Abstract

The main purpose of this study is to design combinational logic gates based on a novel configuration of insulator–metal–insulator (IMI) nanoring plasmonic waveguides. Plasmonic logic gates are half adder, full adder, half subtractor, full subtractor, and one-bit comparator and are realized in one structure. The performance of the logic circuits is based on constructive and destructive interferences between the input and control signals. The transmission threshold value is assumed to be 0.35 at the resonance wavelength of 1.310 μm. The transmission spectrum, contrast loss (CL), insertion loss (IL), modulation depth (MD), and contrast ratio (CR) are calculated in order to evaluate the structure’s performance. The maximum transmission of the proposed structure is 232% for full a adder logic gate, and MD exceeds 90% in all plasmonic combinational logic circuits. The suggested design plays a key role in the photonic circuits and nanocircuits for all-optical systems and optical communication systems. The combinational logic gates are analyzed and simulated using the finite element method (FEM).

## 1. Introduction

In recent years, surface plasmon polaritons (SPPs)-based all-optical devices have been of interest of researchers [1,2,3,4,5,6,7,8]. All-optical SPP devices overcome the diffraction limit in photonic devices and limitations of semiconductor-based electrical devices such as intrinsic delay and significant thermal production. Therefore, in SPP devices, the light can be controlled in a scale smaller than the operational wavelength (subwavelength scale) [3]. Constructive or destructive interference between two or more light signals within two or more waveguides is the principle for controlling the operation of these circuits [5,6,7]. Constructive and destructive interferences are used for switching operations in SPP devices [8]. Subwavelength processors include plasmonic NOT, AND, NAND, OR, NOR, XNOR, and XOR logic gates as their building blocks [2]. Recently, numerous optical plasmonic devices have been proposed using micro/nanoscale logic gates [9,10,11,12,13]. Many all-optical studies have been conducted on combinational logic circuits [14,15,16,17,18,19,20,21,22,23]. Some of these studies have utilized SPPs for designing the combinational logic circuits [14,15,16,17,18,19].

A one-bit comparator using a MZI (Mach-Zehnder Interferometer) and two linear control waveguides has been designed [17]. On-chip half and full adders with a high CR, ultra-compact dimensions, and low threshold power, based on nonlinear plasmonic nanocavities have been experimentally developed [18]. Four optical combinational circuits with high transmission values based on square-shaped resonators and hybrid plasmonic waveguides have been introduced [24]. One-bit and two-bit comparators with high CR levels using two structures based on graphene waveguides have been designed [25]. The one-bit and two-bit structures have footprints of 0.42 µm^2^ and 0.9 µm^2^, respectively. In [26] a structure based on a MIM waveguide and having a footprint of 66 µm^2^ was suggested for two combinational circuits. A pair of combinational logic functions that employed an IMI structure using an elliptical resonator with a size of 540 nm × 250 nm was suggested in [27]. Two combinational circuits with hybrid plasmonic waveguides, the high transmission, high MD, and a 1300 × 400 nm footprint were proposed [28].

In this study, plasmonic combinational logic gates including half adder, full adder, half subtractor, full subtractor, and one-bit comparator were designed in a single IMI-based compact structure. Compared to MIM plasmonic waveguides, the IMI plasmonic waveguides have the advantages of reduced coupling losses, manufacturing simplicity [29], and a relatively higher quality factor [30,31].

Constructive and destructive interference between the signals control the status of the output logic state. The performance of the structure was evaluated by the transmission spectrum, CL, insertion loss IL, MD, and CR. Maximum transmission at the resonance wavelength of 1.310 μm was achieved in full adder mode. The MD parameter was remarkably high in all combinational logic circuits; thus, the structure has been designed with optimal structural parameters. According to the results, the proposed structure may be considered to be a promising candidate for nanophotonic integrated circuits due to its high CR. The designed structure was illuminated by a plane wave with transverse magnetic (TM) polarization. A two-dimensional (2D) configuration within COMSOL Multiphysics software was employed and FEM was utilized to solve Maxwell’s equations and obtain the results.

## 2. The Resonator, Structure Type, and Design Parameter Selection

**Figure 1:**
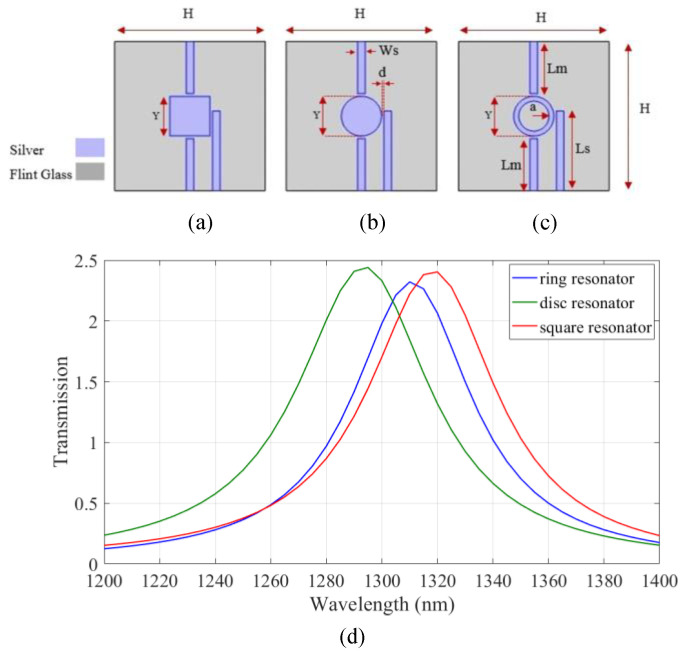
The IMI structures with (**a**) square, (**b**) disk, and (**c**) nanoring resonators and (**d**) transmission spectrum of the devices.

In order to design an all-arithmetic logic unit with high transmission at a wavelength of 1.310 µm, transmission spectra of the three basic IMI structures involving three strips and one resonator are compared in Figure 1, where the selected metal and insulator are silver and flint glass, respectively. Johnson and Christy’s data are used for the permittivity of the silver in the simulation of this proposed design [32].

Flint glass with a refractive index of 1.8 was considered [33,34]. The IMI structures with square, disk, and nanoring resonators are shown in Figure 1a–c, respectively. The geometrical parameters of the devices are listed in Table 1. The transmission spectrum of the devices is shown in Figure 1d. As shown, two input waves are injected and coupled to the lower two strips and the output wave is extracted from the higher strip. According to the results, maximum transmission is achieved using the IMI structure with a nanoring resonator. Therefore, the all-arithmetic logic unit based on nanoring resonator was designed.

**Figure 2:**
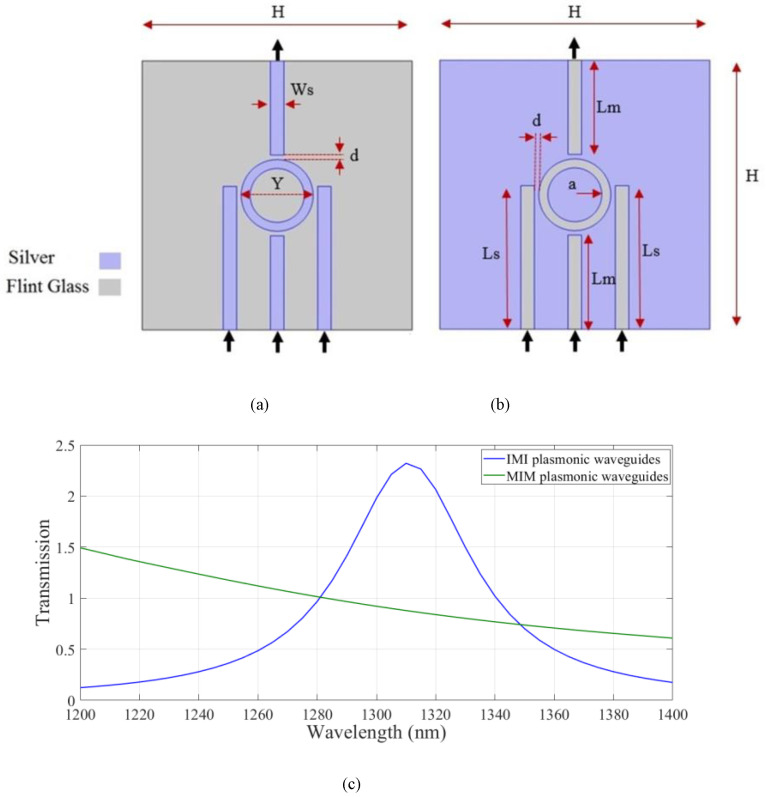
The (**a**) IMI and (**b**) MIM plasmonic structures with nanoring plasmonic resonators and (**c**) transmission spectra of the structures.

In the next step, the transmission spectra of IMI and MIM plasmonic waveguides which possess four strips and one nano ring were compared. The IMI and MIM plasmonic structures are shown in Figure 2a,b, respectively. The structural dimensions of the strips and resonators were the same as those assumed in Figure 1. As shown, the input waves are coupled to the devices through the lower stripes. The output light is extracted from the higher strip. The metal and insulator region are assumed to be silver and flint glass. Transmission spectra of the IMI and MIM waveguides are shown in Figure 2c.

**Figure 3:**
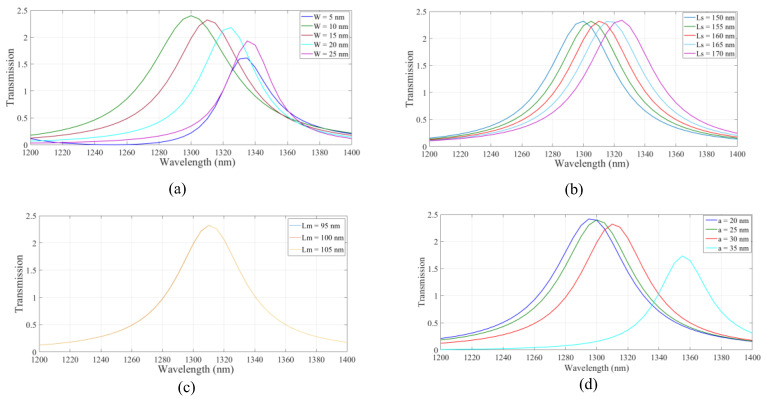
The effect of structural parameters of (**a**) the width of stripes, (**b**) the length of side stripes, (**c**) the length of middle stripes, and (**d**) the nano ring inner radius on the transmission spectrum of the structure.

As shown the structure based on IMI plasmonic waveguides has the higher transmission value at the wavelength of 1.310 µm. Therefore, the IMI-based waveguides were considered hereafter. Next, the effect of changing the geometrical parameters on the transmission spectrum of the IMI plasmonic waveguides was studied. The width of the strips, W_s_, the length of the side stripes, L_s_, the length of the middle strip, L_w_, the inner radius of the nanoring, a, and the distance between the resonator and strips, d, were considered. In Figure 3a, the width of the stripes, W, was changed from 5 nm to 25 nm in increments of 5 nm. The maximum transmission at the desired wavelength was obtained when W was 15 nm. In Figure 3b, the length of the side stripes, L_s_, is swept from 150 nm to 170 nm for stripes with a width of 15 nm. As shown, the resonance wavelength was shifted to the longer wavelength range. The maximum value of transmission was achieved when L_s_ was set to 160 nm. Transmission spectra of the structure for different middle strip lengths of 95, 100, and 105 nm are shown in Figure 3c, where the w and L_s_ parameters are set to 15 nm and 160 nm, respectively. According to the results, there is no change in the transmission spectrum by changing the middle strip length. For simplicity of the structure, a middle strip with a length of 105 nm was considered. Therefore, the distance between all stripes and the resonator was 5 nm. By considering the desired parameters, the effect of the nanoring inner radius on transmission is studied in Figure 3d. Inner radii of 20, 25, 30, and 35 nm were considered. The transmission spectrum experienced red shift when increasing the inner radius of the nanoring. The maximum value of the transmission was obtained at the proper wavelength when a was set to 30 nm. The proper geometrical parameters were used hereafter.

## 3. Structure Layout and Theoretical Model

**Figure 4:**
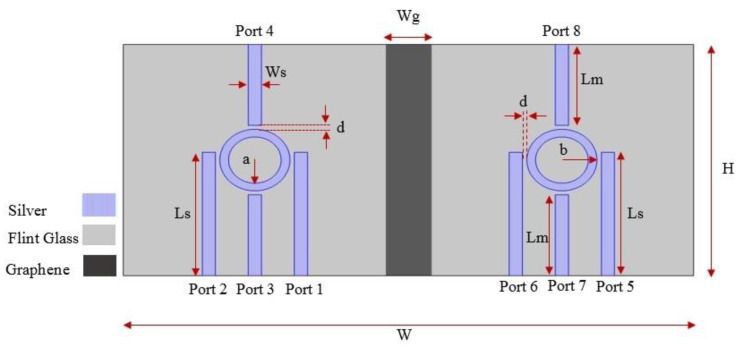
The suggested structure for the plasmonic combinational logic gates.

The proposed structure for the plasmonic combinational logic gates is shown in Figure 4. The structure includes two substructures, including the designed IMI structure in Figure 4a with proper structural parameters. The size of both substructures is 300 nm × 300 nm. The substructures are separated by a perfect electrical conductor (PEC) with a thickness of 50 nm for complete isolation of the substructures. Graphene material may be used as a PEC [35,36].

Table 2 includes structural dimensions of the suggested plasmonic combinational logic gate. The total height and width of the suggested device are 300 nm and 650 nm, respectively. In practice, the third dimension is considered with a value greater than twice the wavelength [37]. The suggested device has a compact size of 300 nm × 650 nm. Optical diffraction occurred in optical components with a size of about the half the wavelength of the light. This limitation affects light propagation as well as the scalability and size of the optical devices [38,39]. This phenomenon can be attributed to the inherent three-dimensional (3D) nature of dielectric wave propagation, as dictated by Equation (1) [4].

(1) $$\beta^{2}+k_{x}^{2}+k_{y}^{2}\;=\;{ɛ}_{c}\frac{\omega^{2}}{c^{2}}$$

where *β* is the propagation constant, *k_x_* is the wave number in x direction, *k_y_* is the wave number in y direction, *ɛ_c_* is the dielectric constant of the core material, *ω* is the angular frequency, and c is the speed of light in free space. In order to effectively couple light into the IMI plasmonic waveguide, light propagation is realized into a dielectric slab waveguide through one of the plasmonic excitation techniques [40,41,42,43]. The dielectric waveguide facilitates the confinement of optical energy and supports the dielectric mode. The efficient transmission of the mode into the IMI plasmonic waveguide is realized with a tapered construction. The taper structure transforms micron-sized dielectric mode into a nano-sized IMI plasmonic mode. The refractive index of the structure in the z-direction is constant; thus, two-dimensional field distributions were considered. The resonance wavelength (*λ_sp_*) can be determined by Equation (2) [44]:

(2) $$\lambda_{\mathrm{sp}}=(4\pi Dn_{eff})$$

where *D* is the larger diameter of the nanoring and *n_eff_* is the effective refractive index.

The resonant wavelength of 1.310 µm is employed in optical communications [45]. The transmission is one of the parameters for measuring the performance of the device. The transmission is defined by the ratio between the output optical power (*Pout*) and a single-input optical power (*Pin*) as given in Equation (3) [46]:

(3) $$T=(\frac{Pout}{Pin})$$

The output state of the desired logic gate is determined by the threshold value of T. In the proposed structure, the threshold value is assumed to be 0.35. Therefore, when T is higher than the threshold value, the output logic state is considered as 1 (ON), and if T is less than the threshold value, the output logic state is considered as logic 0 (OFF). On the other hand, the CR, MD, IL, and CL are utilized to describe device performance. The CR is defined as Equation (4) [47]:

(4) $$\mathrm{CR}\;(\mathrm{dB})=10\log(\frac{Pout|ON_{min}}{Pout|OFF_{max}})$$

A description of the CR’s value is provided in [43]. The MD is defined as the relationship between the maximum level of the transmission in the ON state, denoted as (*MaxT_ON_*) and the minimum level of the transmission in the OFF state, denoted as (*MinT_OFF_*). The MD parameter is obtained as in Equation (5) [48]:

(5) $$\mathrm{Modulation}\;\mathrm{depth}\;(\mathrm{MD})=(\frac{MaxT_{ON}-MinT_{OFF}}{MaxT_{ON}})$$

The ratio determines whether the chosen dimensions for the proposed design are optimal [32]. Insertion loss (IL) is another parameter that demonstrates the relationship between minimal output power in the ON state and input power. IL is defined by Equation (6) [49]:

(6) $$\mathrm{IL}\;(\mathrm{dB})=-10\log(\frac{Pout|ON_{min}}{Pin})$$

The parameter measures the losses caused by the insertion of one device into another. CL quantifies the losses caused by the CR; when the CR is high and insertion loss is low, the induced losses are low, and vice versa. According to Equation (7), the total losses are low whenever the CL is high.

CL (dB) = CR (dB) − IL (dB)
(7)

The proposed structure operates based on constructive and destructive interference between the control signal and the input signal(s).The constructive and destructive interference between the control and the input(s) signal is dependent on the position of the control and input ports and the phase of the incident light signal, where the structural parameters, material, and the shape are not changed. The position of the control port and input port(s) determines the form of interference.

The constructive and destructive interference between the control signal and the input(s) is dependent on the position of the control and input ports and the phase of the incident light signal; the structural parameters, material, and shape remain constant. The position of the control port and input port(s) determines the form of interference. Constructive interference occurs when the phase of the launched wave is the same across all ports, including the control port, and aligns with the propagation direction.

In contrast, destructive interference occurs if the direction of propagation or the phase of the launched wave at each port is different. Equation (8) [46] describes the destructive and constructive interference between incident light signals:

(8) $$m=\left(\frac{\left(4n_{eff}d\cos\theta \right)}{\lambda }\right)$$

where *n_eff_* is the effective refractive index of the silver and m is the interference order as a positive integer greater than zero. *θ* represents the phase of the incident wave, while *λ* represents the incident wavelength. When *θ* = 0°, the sign of Equation (7) is positive. This shows that the modes propagate along the same paths; thus, constructive interference arises between modes with identical phases, leading to an enhancement in transmission. When *θ* = 45°, m has a positive sign and the mode direction aligns with the direction of wave propagation. Therefore, constructive interference occurs between modes with the same phases. The magnitude of the interference mode is smaller than that of *θ* = 0°. Therefore, there is a slight improvement in transmission. When *θ* = 90° is 90, Equation (7) results in zero, indicating the absence of both constructive and destructive interference in the modes.

Transmission is linked to the phases of input and control light waves. When *θ* = 180°, the sign of Equation (7) is negative. This indicates that the modes propagate in opposite directions. Consequently, destructive interference occurs between modes with different phases. Therefore, transmission is decreased.

## 4. The Suggested All-Optical Combinational Logic Circuits

For all suggested plasmonic combinational logic circuits, the structure is excited by a plane wave with a wavelength range of 1000 to 1800 nm. A plane wave with TM polarization is injected into the input and control ports. To solve Maxwell’s equations, COMSOL Multiphysics software was used. The results were obtained by the FEM.

### 4.1. Plasmonic Half Adder Logic Circuit

**Figure 5:**
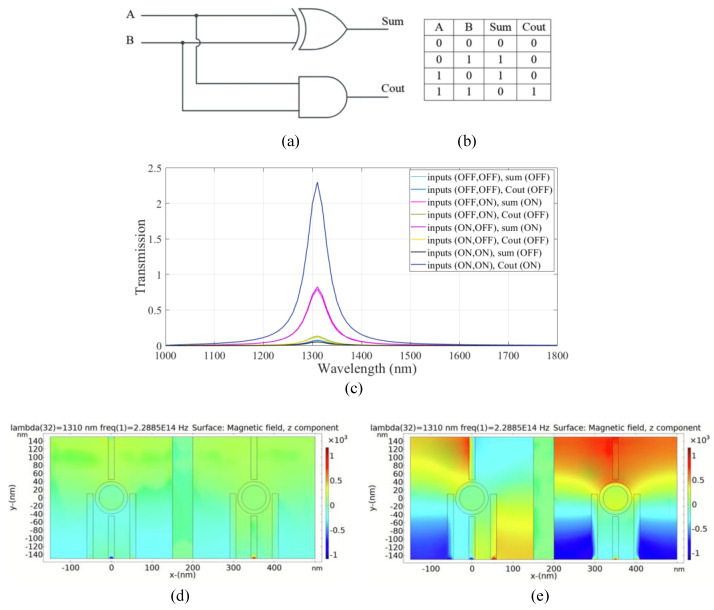
(**a**) The schematics and (**b**) the truth table of the half adder circuit, (**c**) transmission spectra, the distribution of the magnetic field of the plasmonic half adder for the logics of (**d**) 00 and (**e**) 11 inputs.

A half adder is a basic electronic circuit employed for binary addition. It accepts two binary inputs, A and B, and generates two outputs: the summation (sum) and carry (C_out_). The half adder is capable of calculating the sum of the inputs, but it is unable to account for any carry generated by prior addition operations. According to the truth table of the half adder, the first output (sum) is approximated using the XOR gate and the second output (C_out_) is obtained using the AND logic gate. The schematics of the half adder circuit and the truth table are shown in Figure 5a,b. In the proposed structure, the left substructure is selected for obtaining the sum output. The right substructure is selected to generate the carry output.

To design XOR logic gate in the left substructure, ports 1 and 2 are input ports denoted as input 1 and input 2, respectively. Ports 3 and 4 are the control port and output port 1 (sum), respectively. To design the AND gate in the right substructure, ports 5 and 6 are input ports denoted as input 3 and input 4, respectively. Ports 7 and 8 are considered to be the control port and output port 2 (C_out_), respectively.

The constructive and destructive interference between the input signal(s) and the control signal in the two substructures is utilized to realize the half adder combinational logic circuit. According to the transmission spectra shown in Figure 5c, when the incident light with a wavelength of 1.310 µm and a phase of 0° (logic 1) is applied to ports 7 and 3 (the control ports), while the state of ports 1, 2, 5, and 6 (input ports) is OFF (logic 0), the transmission value is 0.07 below the threshold value of 0.35 and the state of ports 4 and 8 (output ports) is OFF (logic 0).

In the following two cases, when the light with a phase of 0° (logic 1) is launched to one of the ports, an input port in the left substructure, while the state of the other port is OFF (logic 0) and the state of port 3 (the control port) is in an ON state (logic 1) with the phase of 0°, constructive interference between the input signal and the control signal occurred and the transmission value in port 4 (sum port) reaches 0.81 (greater than the threshold transmission value). Therefore, the state of port 4 is ON (logic1).

Also, when one of the input ports, port 3 or port 4, in the right substructure, is in an ON state (logic 1) with a phase of 45° and port 7 (the control port) remains ON with a phase of 180°, destructive interference between the signals of the input port and the control port occurs and the transmission value of port 8 (the carry port) is 0.1 and its state is OFF.

In the last state, all ports in the two substructures are exposed to light waves with a different phase for each port. In the left substructure, port 1 and port 2 (the input ports) are in phases of 180° and 45°, respectively. Port 3 (the control port) is in a phase of 0°. Due to the phase mismatch between the signals, destructive interference occurs between the signals and the transmission value in port 4 (the sum port) is equal to 0.05, meaning that port 4 (output port 1) is OFF. In the right substructure, the phase for all ports (the two input ports and the control port) is equal to 180°. In this case, the constructive interference occurs between the signals and the transmission value in port 8 (the carry port) is 2.23, and the state of port 8 (output port 2) is ON. Figure 5d,e shows the distribution of the magnetic field of the plasmonic half adder for the logics 00 and 11 inputs, respectively. The simulation results are summarized in Table 3.

The combinational logic circuit provides a medium CR, which indicates efficient circuit performance [4]. The MD is remarkably high (97.8%), indicating an excellent design with optimal dimensions [32] and very good IL. Finally, a moderate CL is obtained in the structure.

### 4.2. Plasmonic Half Subtractor Logic Circuit

**Figure 6:**
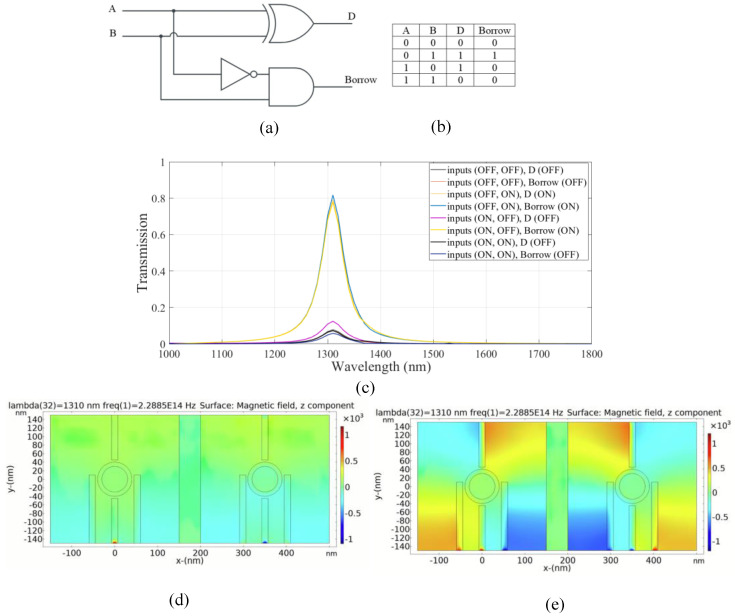
(**a**) The schematics and (**b**) the truth table of the half subtractor circuit, (**c**) transmission spectra, the distribution of the magnetic field of the plasmonic half subtractor for the logics of (**d**) 00 and (**e**) 11 inputs.

A half subtractor is a type of combinational logic circuit consisting of two inputs and two outputs. These outputs are referred to as the difference (D) and the borrow (B). The D output is produced by an XOR gate and the B output is obtained via a logic configuration indicated as *A′B*, as can be seen in Figure 6a,b. The left and right substructures are designed to achieve D and B outputs, respectively. For the D output, the structure is the same as that used for sum output in the half adder. For the B output, port 5 and ports 3, 6 and 7 are considered to be the input port, input port 4, the output port and the control port, respectively.

According to the truth table, B output follows an XOR gate output, but in the third case, when the input states are ON-OFF, the output is in the OFF state. This state is achieved by applying the light to port 2 (input port 2) with a phase of 180° and to the control port with a phase of 0° consistently. As shown in Figure 6c, the transmission value at the resonance wavelength is 0.18 and the output port state is OFF.

Figure 6d,e illustrates the distribution of the magnetic field of the plasmonic half subtractor for the logic 00 and 11 inputs, respectively. The simulation results are provided in Table 4.

According to the results in Table 4, the combinational logic circuit has a medium CR; thus, circuit performance is favorable and efficient [4]. The MD is remarkably high which indicates an excellent design with optimal dimensions [32]. In addition, a low IL and moderate CL are achieved.

### 4.3. Plasmonic Full Adder Logic Circuit

**Figure 7:**
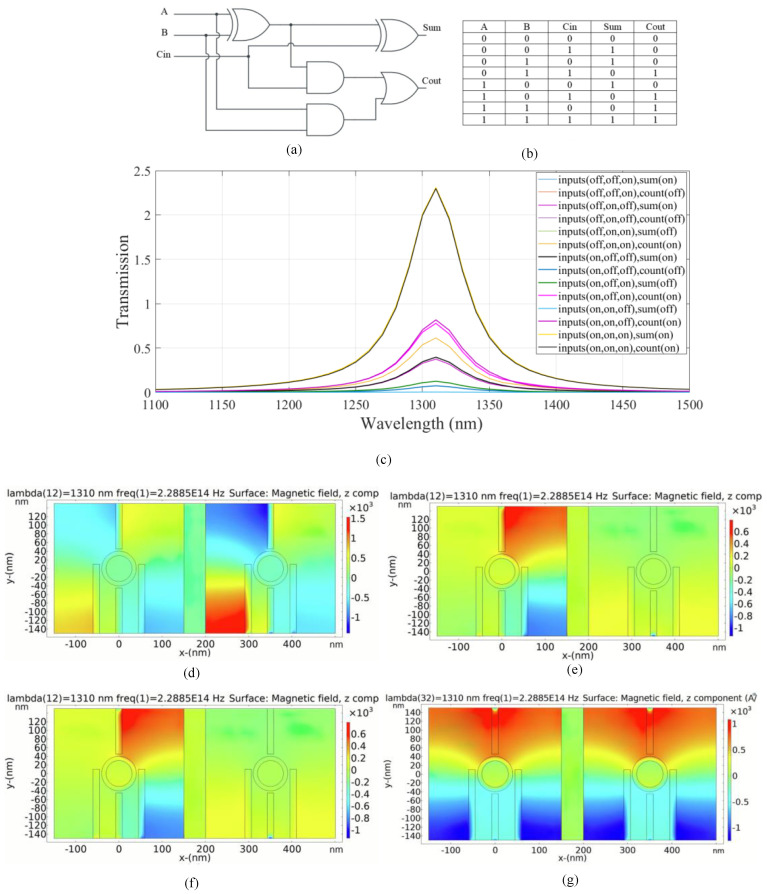
(**a**) The schematics and (**b**) the truth table of the full adder circuit, (**c**) transmission spectra, the distribution of the magnetic field of the plasmonic full adder for the logics of (**d**) 011, (**e**) 100, (**f**) 101, and (**g**) 111 inputs.

A schematic of a full adder logic circuit with the truth table is shown in Figure 7a,b. The full adder considers three input bits including A, B and carry (C_in_) [50]. The left substructure is utilized to compute (sum), the output sum, in the full adder combinational logic circuit, while the right substructure is considered to calculate (C_out_), the output carry.

The transmission spectrum of the full adder is shown in Figure 7c. According to the transmission curves, if one of the input ports is in the ON state and the C_in_ is in the OFF state, only the output sum is in the ON state. The transmission peak value is 0.39 in this case. The peak value of the transmission spectrum for the C_out_ is 0.07 related to the OFF logic state. According to the truth table and transmission spectrum, the C_out_ is in the ON state in the fourth case and the transmission value of 1.55 is achieved. The output sum remains in the OFF state and the transmission value of 0.003 is below the transmission threshold. For the sixth and seventh cases, the carry output is activated with transmission values of 0.77 and 0.8, respectively. On the other hand, the output sum remains in the OFF state and the transmission values are 0.12 and 0.002, respectively. In the eighth state, both outputs are activated with an ON state and the transmission value is 2.3. Constructive interference occurs when the input signals have identical phases. Destructive interference occurs when the phases of the input signals are different.

Figure 7d–g indicates the distribution of magnetic field of the logics of 011, 100, 101, and 111 inputs in the plasmonic full adder, respectively. Table 5 provides a summary of the simulation results of the suggested plasmonic full adder combinational logic circuit.

Based on the results in Table 5, the combinational logic circuit has a moderate CR which indicates efficient circuit performance [4]. The MD is remarkably high (99.8%); thus, an excellent design with optimal dimensions is completed [32]. An acceptable IL is calculated. The circuit has an acceptable CL.

### 4.4. Plasmonic Full Subtractor Logic Circuit

**Figure 8:**
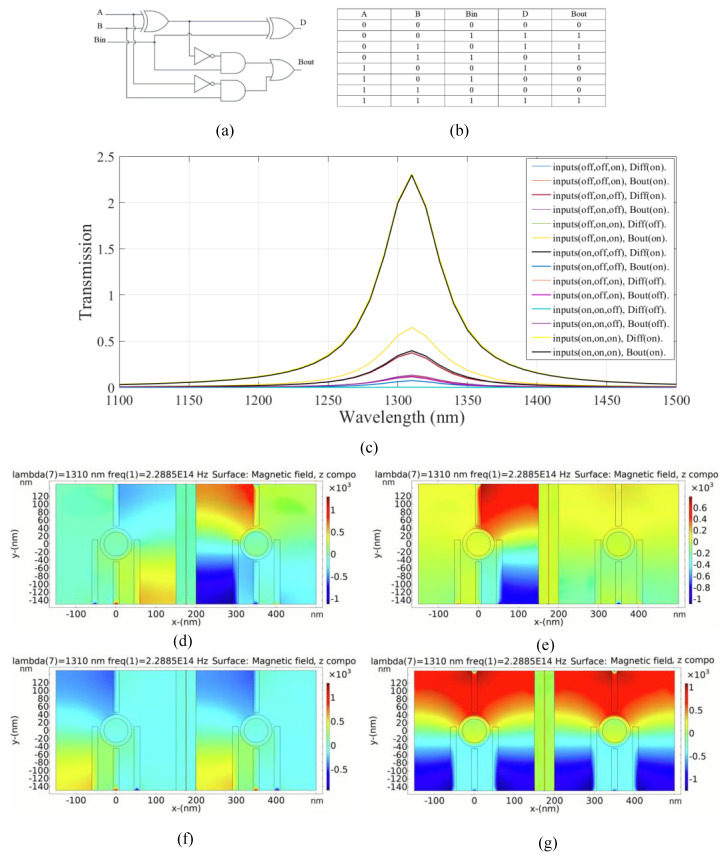
(**a**) The schematics and (**b**) the truth table of the full subtractor circuit, (**c**) transmission spectra, the distribution of the magnetic field of the plasmonic full subtractor for the logics of (**d**) 001, (**e**) 011, (**f**) 110, and (**g**) 111 inputs.

A full subtractor is a combinational logic circuit that requires three inputs: the minuend (A), the subtrahend (B), and the borrow-in (Bin), and produces two outputs: the difference (D) and the borrow out (Bout). A schematic of the full subtractor with truth table is shown in Figure 8a,b.

The left substructure is utilized to compute the output difference (first output) in the full-subtractor combinational logic circuit. Additionally, the right substructure is employed to calculate the output borrow (second output). This combinational logic circuit operates through the constructive and destructive interference of input signals within each substructure.

The transmission of the full subtractor for different states of the inputs is shown in Figure 8c. In the first and second cases, the output difference is in the ON state according to the transmission values of 0.39 and 0.37, respectively. In the fourth state, however, only the output borrow is in the ON state and the transmission value exceeds 0.6; while the state of the output difference is OFF. In the fifth state, only the output difference is in the ON state, based on the transmission value of 0.39. The output borrow is in the OFF state. In this case, the transmission at the resonance wavelength is 0.07. For two consecutive cases, the transmission values of the output difference are 0.12 and 0.002, respectively. The output difference remains in the OFF state because the transmission values are below the transmission threshold. The output borrow is also in the OFF state and the transmission peak values of 0.11 and 0.13 are obtained, respectively. In the eighth state, both outputs are activated and in the ON state. The transmission peak exceeds 2.3.

Additionally, Figure 8d–g demonstrates the distribution of magnetic field of the logic 001, 011, 110, and 111 inputs in the proposed plasmonic full subtractor, respectively. Table 6 provides a summary of the results of the proposed plasmonic full subtractor combinational logic circuit.

As shown in Table 6, the combinational logic circuit has a moderate CR [4]. The MD is remarkably high (99.91%); thus, an excellent design with optimal dimensions is carried out [32]. The circuit has acceptable IL. Finally, the circuit achieves an acceptable CL.

### 4.5. Plasmonic One Bit Comparator Logic Circuit

**Figure 9:**
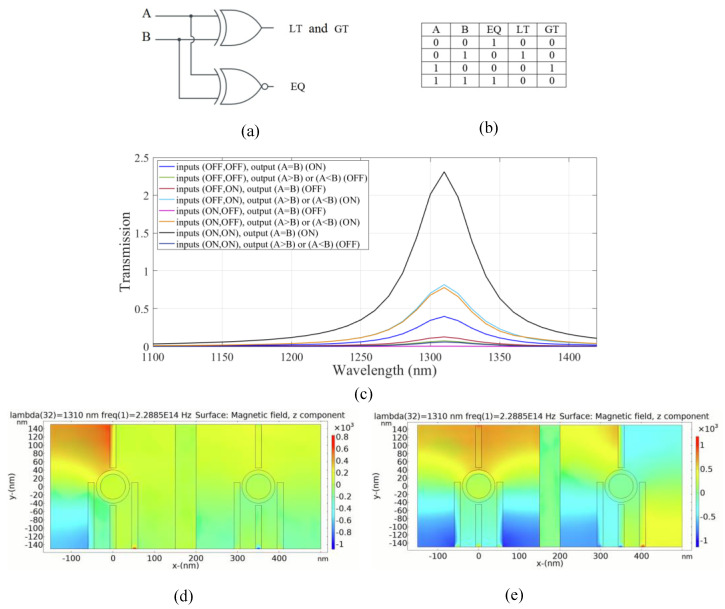
(**a**) The schematics and (**b**) the truth table of the full one-bit comparator circuit, (**c**) transmission spectra, the distribution of the magnetic field of the plasmonic one-bit comparator for the logics (**d**) 00, (**e**) 11 inputs.

A one-bit comparator is a combinational logic circuit that compares two bits and determines their relative magnitudes. It receives a pair of input bits, namely A and B, and generates three output signals namely equal (EQ), greater than (GT), and less than (LT). The output signal for equality (EQ) is represented by an XNOR gate, while the second and third output signals are combined and represented by an XOR gate, as shown in Figure 9a. The truth table of the circuit is shown in Figure 9b.

In our structure, the left substructure is employed for EQ operation. On the other hand, the right substructure is utilized for inequality operations, GT and LT outputs.

In the left substructure, to achieve an XNOR logic gate, the input ports 1 and 2 are considered to be ports 2 and 3, respectively. Port 1 is assumed to be the control port, and the output port 1 (EQ) is considered to be port 4. For the right substructure, port 5, port 6, port 8, and port 7 are considered to be input port 3, input port 4, output port 2 and the control port, respectively.

The inequality operation of the combinational logic circuit is similar to the sum operation in a half adder and the difference operation in a half subtractor. In the proposed plasmonic inequality circuit, the output represents the functions of “LT” (less than) or “A < B” in the second case, and “GT” (greater than) or “A > B” in the third case.

The transmission spectrum of the circuit is shown in Figure 9c. For achieving equality output, the XNOR gate is realized in the left substructure. By applying light with a wavelength of 1.310 µm wavelength and a phase of 180° to the control port, the output port 1 (EQ) is in the ON state and the transmission value exceeds the threshold of 0.35. In the second and third cases, the destructive interference between the input signal and control signal occurs due to the phase difference. Consequently, the transmission value is below the threshold and the output is in the logic 0. In the fourth state, there is a substantial constructive interference between the control signal and the input signals with the phase of 180°. Therefore, the transmission is significantly enhanced by 232% and the output is in the logic 1 state. Thus, the desired operation of the one-bit comparator combinational logic circuit is successfully accomplished.

Figure 9d,e depicts the magnetic field distribution of the logic 00 and 11 input in the plasmonic one-bit comparator, respectively. The simulation results of the suggested plasmonic one-bit comparator are summarized in Table 7.

According to the results in Table 7, the combinational logic circuit provides a moderate CR [4]. The MD is remarkably high (99.8%); thus, an excellent design with optimal dimensions is completed [32]. The circuit has a moderate IL and an acceptable CL.

### 4.6. Comparing the New Work with Previous Research Efforts

A comparison between the suggested plasmonic combinational logic circuit and the other previous works is listed in Table 8.

## 5. Conclusions

In this paper, five combinational logic gates based on IMI nanoring plasmonic waveguides are designed. These combinational logic gates, including a half adder, full adder, half subtractor, full subtractor, and one-bit comparator are realized by a single structure. The transmission spectrum, CL, IL, MD, and CR are calculated for all logic gates. The transmission value can be controlled by the position and the phase of input and control ports of the structure. The transmission threshold value is assumed to be 0.35 at the wavelength of 1.310 μm to determine the logic states of the outputs. The transmission of the designed structure is 232% in the full adder logic gate. The MD value of above 90% is obtained for all logic gates. FEM is used to simulate and analyze the proposed structure.

## Figures and Tables

**Table 1:** The geometrical parameters of the devices shown in Figure 1a–c.

Parameter	Description	Value (nm)
H	Height and width of the structure	300
L_s_	Length of the side stripes	160
L_m_	Length of the middle stripes	105
Y	Side length of the nano square resonator, Diameter of the nanoring resonator	80
a	Nanoring inner radius	30
W_s_	Width of the stripes	15
d	Distance between the stripes and resonator	5

**Table 2:** Structural parameters of the designed plasmonic combinational logic gate.

Parameter	Description	Value (nm)
H	Height of the structure	300
W	Width of the structure	650
Ls	Length of the side stripes	160
Lm	Length of the middle stripes	105
Wg	Graphene layer width	50
b	Nanoring outer radius	40
a	Nanoring inner radius	30
Ws	Width of the stripes	15
d	Distance between the stripes and nanoring	5

**Table 3:** The summary of the simulation results of the half adder circuit.

	Input Port 1	Cont.1	Input Port 2	Sum (T)	Input Port 3	Cont.2	Cout (T)	CR (dB)	MD	CL (dB)	IL (dB)
Ports number	1	3	2	4	5	7	8	8.67	97.8%	0.9	7.77
Ports statue	0	ON (0°)	0	OFF (0.07)	0	ON (180°)	OFF (0.07)				
	0	ON (0°)	1 (0°)	ON (0.81)	0	ON (180°)	OFF (0.11)				
	1 (0°)	ON (0°)	0	ON (0.81)	1 (45°)	ON (180°)	OFF (0.11)				
	1 (180°)	ON (0°)	1 (45°)	OFF (0.05)	1 (180°)	ON (180°)	ON (2.32)				

**Table 4:** The simulated transmission values for the suggested plasmonic half subtractor.

	Input Port 1	Input Port 2	Cont.1	D (T)	Input Port 4	Input Port 3	Cont.2	Borrow (T)	CR (dB)	MD	CL (dB)	IL (dB)
Ports number	1	2	3	4	6	5	7	8	8.1	93%	7.08	1.02
Ports statue	0	0	1 (180°)	OFF (0.077)	0	0	1 (0°)	OFF (0.07)				
	0	1 (180°)	1 (180°)	ON (0.79)	1 (0°)	0	1 (0°)	ON (0.81)				
	1 (45°)	0	1 (180°)	OFF (0.12)	0	1 (0°)	1 (0°)	ON (0.81)				
	1 (45°)	1 (180°)	1 (180°)	OFF (0.072)	1 (45°)	1 (180°)	1 (0°)	OFF (0.05)				

**Table 5:** The simulated transmission values for the suggested plasmonic full adder.

	Input Port 1	Input Port 2	Cin Port	Sum (T)	Input Port 3	Input Port 4	Cin Port	Cout (T)	CR (dB)	MD	CL (dB)	IL (dB)
Ports number	3	2	1	4	5	6	7	8	4.8	99.8%	4.3	0.5
Ports statue	0	0	0	OFF	0	0	0	0				
	0	0	1 (180°)	ON (0.39)	0	0	1 (0°)	OFF (0.073)				
Ports number	3	2	1	4	6	7	5	8				
Ports statue	0	1 (0°)	0	ON (0.37)	0	1 (0°)	0	OFF (0.073)				
Ports number	3	2	1	4	7	6	5	8				
Ports statue	0	1 (180°)	1 (45°)	OFF (0.0039)	0	1 (0°)	1 (0°)	ON (1.55)				
Ports number	1	2	3	4	7	6	5	8				
Ports statue	1 (0°)	0	0	ON (0.39)	1 (0°)	0	0	OFF (0.07)				
Ports number	3	2	1	4	7	6	5	8				
	1 (45°)	0	1 (180°)	OFF (0.12)	1 (0°)	0	1 (0°)	ON (0.77)				
	1 (180°)	1 (45°)	0	OFF (0.11)	1 (0°)	1 (0°)	0	ON (0.8)				
	1 (0°)	1 (0°)	1 (0°)	0 (2.3)	1 (0°)	1 (0°)	1 (0°)	ON (2.3)				

**Table 6:** The simulated transmission values for the suggested plasmonic full subtractor.

	Input 1	Input 2	Input 3 (Bin)	D (T)	Input 1	Input 2	Input 3 (Bin)	Bout (T)	CR (dB)	MD	CL (dB)	IL (dB)
Ports number	3	2	1	4	7	6	5	8	4.89	99.91%	0.59	4.3
Ports statue	0	0	0	OFF	0	0	0	OFF				
	0	0	1 (0°)	ON (0.39)	0	0	1 (0°)	ON (0.37)				
	0	1 (0°)	0	ON (0.37)	0	1 (0°)	0	ON (0.4)				
Ports number	1	3	2	4	5	7	6	8				
Ports statue	0	1 (180°)	1 (45°)	OFF (0.11)	0	1 (0°)	1 (45°)	ON (0.6)				
Ports number	1	2	3	4	7	6	5	8				
Ports statue	1 (0°)	0	0	ON (0.39)	1 (0°)	0	0	OFF (0.07)				
	1 (45°)	0	1 (180°)	OFF (0.12)	1 (180°)	0	1 (45°)	OFF (0.11)				
	1 (180°)	1 (45°)	0	OFF (0.002)	1 (45°)	1 (180°)	0	OFF (0.13)				
	1 (0°)	1 (0°)	1 (0°)	ON (2.3)	1 (0°)	1 (0°)	1 (0°)	ON (2.29)				

**Table 7:** The simulated transmission values for the suggested plasmonic one-bit comparator.

	Cont.1	Input Port 1	Input Port 2	Output Port A = B	Input Port 3	Input Port 4	Cont. 2	Output Port A > B or A < B (T)	CR (dB)	MD	IL (dB)	CL (dB)
Ports number	1	2	3	4	5	6	7	8	5.49	99.87%	4.08	1.41
Ports statue	ON (180°)	0	0	ON (0.39)	0	0	ON (0°)	OFF (0.07)				
	ON (180°)	0	1 (45°)	0FF (0.11)	0	1 (0°)	ON (0°)	ON (0.81)				
	ON (180°)	1 (45°)	0	0FF (0.003)	1 (0°)	0	ON (0°)	ON (0.77)				
	ON (180°)	1 (180°)	1 (180°)	ON (2.32)	1 (180°)	1 (45°)	ON (0°)	OFF (0.05)				

**Table 8:** Comparison between the suggested plasmonic combinational logic circuit and the other previous works.

References	Used Software	Number of Combinational Circuits	Proposed Combinational Function	Size	Operating Wavelength	Complexity	Performance Parameters
[24]	FEM-2D	4-comb. Logic circuits	Half Adder, Half Subtractor, Full Adder, 4-bit converter	850 nm × 400 nm 1750 nm × 400 nm	1310 nm	more	T, CR, MD, IL
[25]	FDTD	2-comb. Logic circuits	One-bit comparator, Two-bit comparator	0.42 µm^2^	15 µm	more	T, CR
[26]	FDTD	2-comb. Logic circuits	Half Adder, Half Subtractor	66 µm^2^	66 µm	more	T, CR, IL
[27]	FEM-2D	2-comb. Logic circuits	Half Adder, Half Subtractor	540 nm × 250 nm	850 nm	more	T, MD, CR, IL
[28]	FEM-2D	2-comb. Logic circuits	2 × 1 Multiplexer, Comparator	400 nm × 400 nm 1300 nm × 400 nm	1310 nm	more	T, CR, MD, IL
[51]	FEM-2D	4-comb. Logic circuits	One-bit comparator, Half Adder, Full Adder, Half Subtractor	850 nm × 400 nm	1550 nm	more	T
This paper	FEM-2D	5-comb. Logic circuits	One-bit comparator, Half Adder, Full Adder, Half Subtractor, Full Subtractor	650 nm × 300 nm	1310 nm	less	T, CR, MD, IL, CL

## Data Availability

The data presented in this study are available upon request from the corresponding author.
